# Case Reports: Three cases of autoimmune glial fibrillary acidic protein astrocytosis in disguise

**DOI:** 10.3389/fimmu.2025.1546372

**Published:** 2025-07-15

**Authors:** Wen Zhao, Luyao Gong, Junbin Wang, Zhiguang Chen, Zhe Li, Qiaozhen Su, Youbi Shen, Chunye Zheng

**Affiliations:** ^1^ The Second School of Clinical Medicine, Guangzhou University of Chinese Medicine, Guangzhou, China; ^2^ Department of Medical Imaging, Guangdong Provincial Hospital of Traditional Chinese Medicine, Guangzhou, China; ^3^ Department of Neurology, Guangdong Provincial Hospital of Traditional Chinese Medicine, Guangzhou, China

**Keywords:** glial fibrillary acidic protein astrocytosis, astrocytes, autoantibodies, autoimmune encephalitis, autoimmune neurology, meningoencephalitis, GFAP

## Abstract

Autoimmune glial fibrillary acidic protein astrocytosis (GFAP-A), a novel inflammatory autoimmune disorder of the central nervous system, manifests with insidious onset and demonstrates protean clinical manifestations, which frequently leads to diagnostic ambiguity in early disease stages. We describe three typical GFAP-A cases exhibiting multisystem neurological involvement. Our observations show that GFAP-A frequently clinically mimics tuberculous meningitis (TBM), autoimmune encephalitis (AE), neuromyelitis optica spectrum disorder (NMOSD), Parkinson’s disease (PD), and other neurological diseases. Notably, isolated ataxia is a rare presentation in GFAP-A, which allows us usually to consider spinocerebellar ataxia (SCA). Currently, no established diagnostic criteria or standard treatment protocols exist for GFAP-A. Patients with GFAP-A respond well to corticosteroid therapy. We found detecting GFAP-IgG in cerebrospinal fluid or serum is essential for differentiation.

## 1 Introduction

Autoimmune glial fibrillary acidic protein astrocytosis (GFAP-A) is a newly identified inflammatory autoimmune disorder of the central nervous system (CNS), first reported by Fang et al. at the Mayo Clinic in 2016 (1). Since GFAP-A often affects the brain, meninges, spinal cord, and optic nerves, it can be easily confused with other neurological diseases during early-stage diagnosis (2). The clinical spectrum of GFAP-A is diverse and includes not only headache, fever, encephalitis, myelitis, and visual abnormalities but also tremors, dementia, ataxia, and autonomic dysfunction (3). The extensive array of radiographic findings and diverse clinical presentations may resemble numerous other causes of encephalopathy (4). It is important to acknowledge the substantial diversity within neuroimmune diseases, encompassing autoimmune demyelination, opportunistic and neurotrophic infections, paraneoplastic syndromes, and neurodegenerative and neuropsychiatric disorders (5). Current diagnostic approaches based on GFAP-IgG detection in the cerebrospinal fluid (CSF) or serum face challenges, as co-occurrence with other antibodies or immunological mimicry may confound interpretation. Through three GFAP-A cases with distinct manifestations, we hope to provide some insights to improve the accuracy of diagnosis.

## 2 Case description

### 2.1 Case 1

**Figure 1:**
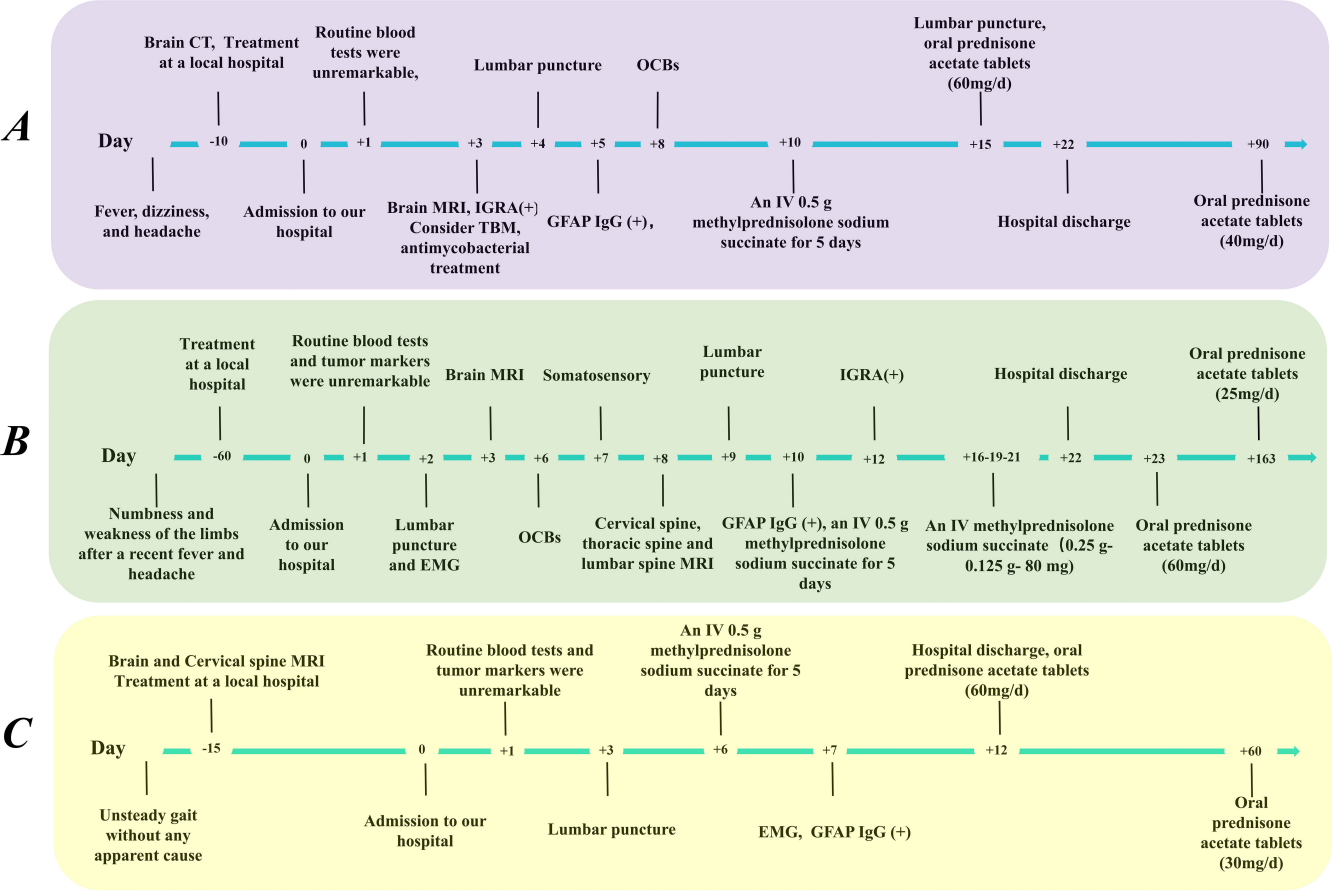
Timeline of clinical manifestations and treatment progression in our patients. **(A)** Patient A, **(B)** Patient B, **(C)** Patient C. CSF, cerebrospinal fluid; CT, computed tomography; EMG, electromyography; IGRA, interferon gamma release assay; IV, intravenous; GFAP, glial fibrillary acidic protein; MRI, magnetic resonance imaging; OCBs, oligoclonal bands.

**Figure 2:**
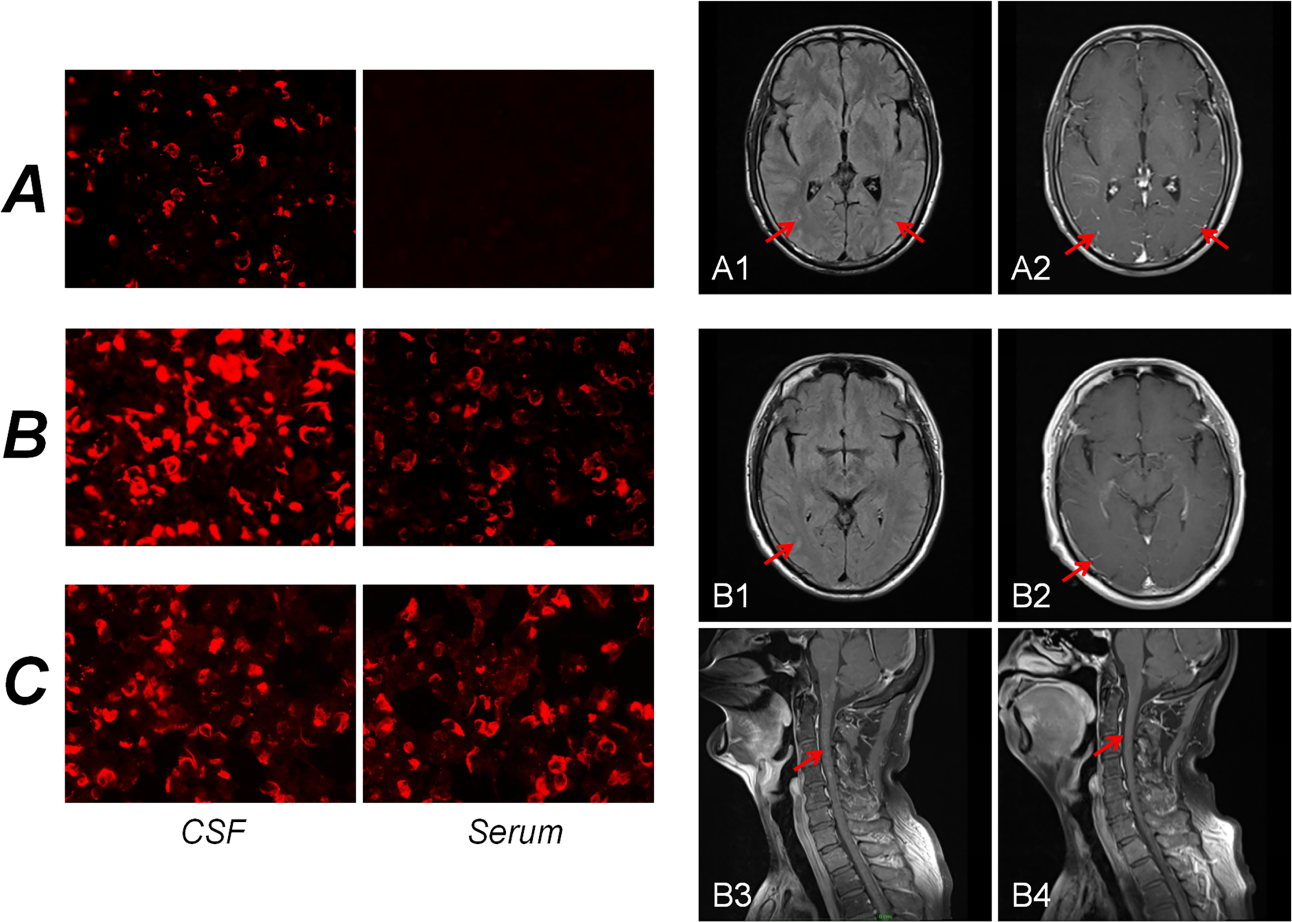
Immunofluorescence pattern of GFAP-IgG in cells transfected with CBA. For Patient A, GFAP-IgG was absent from the serum but present in the CSF **(A)**. For Patient B, GFAP-IgG was also present in both the serum and CSF, with a significantly higher titer observed in the CSF than in the serum **(B)**. For Patient C, GFAP-IgG was detected in both the serum and CSF **(C)**. Brain MR findings of the patients. For Patient A, the T2-FLAIR sequence demonstrated slightly elevated signal intensity in the bilateral occipital lobes and cerebellar hemispheres **(A1)**. Concurrently, contrast-enhanced T1-weighted images revealed leptomeningeal enhancement **(A2)**. For Patient B, the T2-FLAIR sequence revealed an uneven, mild increase in signal intensity within the bilateral occipital occipital sulci **(B1)**. However, the contrast-enhanced T1-weighted images showed no abnormal enhancement **(B2)**. Notably, patchy abnormal enhancement of the spinal cord was observed on contrast-enhanced T1-weighted images **(B3)**, which subsequently resolved after treatment **(B4)**. CBA, cell-based assay; CSF, cerebrospinal fluid.

A 36-year-old male (Patient A) presented with severe fever, dizziness, and headache for 10 days. Initial empirical antimicrobial therapy failed to alleviate symptoms, prompting hospitalization (**Figure 1A**). The first diagnostic lumbar puncture demonstrated elevated CSF pressure (230 mm H_2_O), increased protein concentration (1.22 g/L), and lymphocytic pleocytosis (98%; **Table 1**). Based on epidemiological considerations and CSF profile, the attending physicians initially suspected tuberculous meningitis (TBM) and initiated standard antitubercular therapy. After the targeted therapy, neurological symptoms persisted. Transfer to our hospital enabled advanced neuroimaging evaluation, demonstrating characteristic bilateral leptomeningeal enhancement in occipital regions and cerebellar hemispheres on gadolinium-enhanced T1-weighted MRI. Repeat CSF analysis had similar results as before, while the serum interferon-gamma release assay (IGRA) test was positive. The diagnostic paradigm shifted after cell-based assay (CBA) identified GFAP-IgG in CSF (**Table 2**; **Figure 2A**). Immediate discontinuation of antitubercular therapy and initiation of pulse methylprednisolone sodium succinate (IV 0.5 g/day for 5 days) followed by oral prednisone taper (60 mg/day) achieved remarkable clinical improvement. Upon discharge after 20 days of hospitalization, the patient reported substantial resolution of dizziness, with no headache, fever, or chills. The modified Rankin Scale (mRS) score also improved from 2 at admission to 0 following treatment. The final diagnosis was established as GFAP-A.

**Table 1:** Serum and CSF marker levels before treatment.

Pt No.	Laboratory findings in serum			Laboratory findings in CSF				
	CRP (0-6.00 mg/L)	Leukocyte count (3.50-9.50 E9/L)	Lymphocyte count (1.10-3.20 E9/L)	Pressure (80~180 mmH2O)	WBC (0-8.00 E6/L)	Neutrophil (0-6%)	Lymphocyte (40-80%)	Protein (0.15~0.45 g/L)
A	39.44	9.45	0.81	230	130	2.00	98.00	1.22
B	1.70	5.73	0.47	200	51	0%	100%	1.80
C	ND	8.81	3.97	150	4	ND	ND	0.33

CRP, C-reactive protein; CSF, cerebrospinal fluid; ND, Not tested; WBC, white blood cell.

**Table 2:** Serum and CSF antibody levels before treatment for cell-based assays.

Antibody	Antigen	Patient A		Patient B		Patient C	
		CSF	Serum	CSF	Serum	CSF	Serum
Group I: Antibodies associated with autoimmune diseases of the central nervous system							
Anti-GFAP	Glial Fibrillary Acidic Protein	1:32*	(-)	1:320*	1:32*	1:32*	1:32*
Anti-AQP4	Aquaporin-4	(-)	(-)	(-)	(-)	(-)	(-)
Anti-MOG	Myelin oligodendrocyte glycoprotein	(-)	(-)	(-)	(-)	(-)	(-)
Anti-MBP	Myelin basic protein	(-)	(-)	(-)	(-)	ND	ND
Anti-GAD65	Glutamic acid decarboxylase	(-)	(-)	(-)	(-)	(-)	(-)
Anti-mGluR1	Metabotropic glutamate receptor 1	(-)	(-)	(-)	(-)	(-)	(-)
Anti-mGluR5	Metabotropic glutamate receptor 5	(-)	(-)	(-)	(-)	(-)	(-)
Anti-NMDAR	N-methyl-D-aspartate receptor subunit NR1	(-)	(-)	ND	ND	(-)	(-)
Anti-LGI1	Leucine-rich glioma-inactivated protein 1	(-)	(-)	ND	ND	(-)	(-)
Anti-GABA_A_R	γ-amino butyric acid type A receptor	(-)	(-)	ND	ND	(-)	(-)
Anti-GABA_B_R	γ-amino butyric acid type B receptor	(-)	(-)	ND	ND	(-)	(-)
Anti-CASPR2	Contactin-associated protein-like 2	(-)	(-)	ND	ND	(-)	(-)
Anti-AMPA1	α-amino-3-hydroxy-5-methyl-4-isoxazolepropionic acid receptor subunits GluA1	(-)	(-)	ND	ND	(-)	(-)
Anti-AMPA2	α-amino-3-hydroxy-5-methyl-4-isoxazolepropionic acid receptor subunits GluA2	(-)	(-)	ND	ND	(-)	(-)
Anti-IgLON5	IgLON family member 5	(-)	(-)	ND	ND	(-)	(-)
Anti-DPPX	Dipeptidyl-peptidase-like protein 6	(-)	(-)	ND	ND	(-)	(-)
Anti-GlyR1	Cytokine-like nuclear factor N-PAC	(-)	(-)	ND	ND	(-)	(-)
Anti-D2R	Dopamine D2 receptor	(-)	(-)	ND	ND	(-)	(-)
Anti-Neurexin-3α	Neurexin-3α	(-)	(-)	ND	ND	(-)	(-)
Anti-KLHL11	Kelch-like protein 11	(-)	(-)	ND	ND	(-)	(-)
Anti-gAChR	Ganglion (α3)-type nicotinic acetylcholine receptor	(-)	(-)	ND	ND	(-)	(-)
Group II: Paraneoplastic syndrome autoimmune antibodies							
Anti-Hu (ANNA-1)	HuD, HuC, HuB	ND	ND	(-)	(-)	(-)	(-)
Anti-Yo (PCA-1)	CDR62/CDR2, CDR34/CDR1	ND	ND	(-)	(-)	(-)	(-)
Anti-Ri (ANNA-2)	Nova-1, Nova-2	ND	ND	(-)	(-)	(-)	(-)
Anti-CRMP5	Collapsin response mediator protein 5	ND	ND	(-)	(-)	(-)	(-)
Anti-Ma1	Ma1	ND	ND	(-)	(-)	(-)	(-)
Anti-Ma2	Ma2	ND	ND	(-)	(-)	(-)	(-)
Anti-Amphiphysin	Amphiphysin	ND	ND	(-)	(-)	(-)	(-)
Anti-DNER	Delta/Notch-like epidermal growth factor-related receptor	ND	ND	(-)	(-)	(-)	(-)
Anti-Sox1	Sry-like high mobility group box protein 1	ND	ND	(-)	(-)	(-)	(-)
Anti-Zic4	Zinc finger protein 4, other Zic proteins	ND	ND	(-)	(-)	(-)	(-)
Anti-GAD65	Glutamic acid decarboxylase	ND	ND	(-)	(-)	(-)	(-)
Anti-Recoverin	Recoverin	ND	ND	(-)	(-)	(-)	(-)
Anti-Titin	Titin	ND	ND	(-)	(-)	(-)	(-)
Anti-PKCγ	Protein kinase C gamma	ND	ND	(-)	(-)	(-)	(-)

(-): Negative (cutoff value: 1:10); *Positive (titer values shown); CSF, cerebrospinal fluid; ND, Not tested.

### 2.2 Case 2

A 56-year-old male (Patient B) presented with a 2-month history of fever, progressive quadri-limb numbness/weakness, and 10 kg weight loss (**Figure 1B**). Initial hypothermia therapy resolved fever but not neurological symptoms, which progressed to include diplopia, limb tremors, and sensory deficits below wrists/ankles with preserved vibration sense. The Expanded Disability Status Scale for Admission (EDSS) scores were as high as 6. MRI revealed pontomedullary and spinal cord gadolinium enhancement (**Figure 2B**). The nerve conduction study (NCS) revealed (1): a 30% amplitude reduction in left median nerve conduction (demyelination pattern); (2) prolonged tibial H-reflexes suggesting lumbosacral radiculopathy; (3) abnormal somatosensory-evoked potentials indicating central sensory pathway dysfunction. CSF analysis revealed elevated protein, leukocyte, and lymphocyte counts, with intrathecal oligoclonal band synthesis (OCBs), and the CSF OCBs count was higher than that in the serum. GFAP-IgG was positive by CBA in CSF and serum, with a higher CSF-to-serum titer ratio (1:320 vs. 1:32), confirming GFAP-A. Both tumor markers—specifically carcinoembryonic antigen (CEA), alpha-fetoprotein (AFP), and carbohydrate antigen 19-9 (CA19-9)—and systemic inflammatory indices were within normal reference ranges. Other autoimmune antibodies about paraneoplastic syndrome were negative in both serum and CSF (**Table 2**). Pulse corticosteroid therapy followed by oral tapering achieved rapid symptom resolution, including improved limb strength, resolved diplopia, and tremor cessation. Follow-up MRI demonstrated partial lesion regression and the EDSS score on the review was reduced to 1.

### 2.3 Case 3

More than half a month ago, a man of 47 years of age (Patient C) began experiencing unsteady gait without any apparent cause, accompanied by weakness in both lower limbs and dizziness (**Figure 1C**). He was taken to a local hospital, and appropriate tests were conducted. Following an MRI scan of the head and genetic molecular testing, no significant abnormalities were found. Gradually, the patient also developed cognitive decline, speech and logical impairments, and difficulty completing fine motor tasks with his hands, prompting him to seek treatment at our hospital. On examination, the patient presented with reduced responsiveness, less fluent speech, unclear articulation, and instability during the bilateral finger-nose and heel-tibia tests. The patient exhibited severe limb ataxia, with an International Cooperative Ataxia Rating Scale (ICARS) score of 52. The CSF analysis showed normal cellularity and proteins, but seropositivity for GFAP-IgG in both CSF and serum (**Tables 1**, **2**; **Figure 2**). Electromyography (EMG) demonstrated demyelinating neuropathy in the left common and superficial peroneal nerve. There were no significant abnormalities in other routine examinations. After steroid therapy (IV 0.5 g/day for 5 days), the patient’s symptoms soon improved, with a more stable and accurate gait, reduced weakness in both lower limbs, and diminished dizziness. The patient’s ICARS score improved significantly to 5 at discharge.

## 3 Discussion

As shown above, all patients presented with subacute disease, with two individuals reporting a history of preceding infections, initially presenting with fever and headache. Core manifestations include meningoencephalitis, meningoencephalomyelitis, and myelitis (6), whereas isolated myelitis is relatively rare (7). Currently, there are no uniform diagnostic criteria or standard treatment protocols for GFAP-A (3). In practice, the diagnosis of GFAP-A is usually based on the patient’s typical symptoms and the detection of GFAP antibodies in CSF and serum. Notably, CSF GFAP-A-IgG shows superior specificity (8). Patients exhibit a favorable response to immunotherapy, including corticosteroids, intravenous immunoglobulin, and immunosuppressants (9). In this study, we specifically report some typical clinical symptoms of 3 different patients with GFAP-A and review the literature concerning different diseases that may masquerade as GFAP-A.

All three typical presentations involved lesions affecting the meninges and spinal cord. In addition, the presence of inflammatory markers in the CSF suggested that CNS infection was the most likely diagnosis. Patient A exhibited symptoms consistent with meningitis and his CSF profile paralleled TBM, while positive IGRA reinforced this misdiagnosis. GFAP-A is frequently mistaken for TBM (10), with previous research indicating a misdiagnosis rate between 4.5% and 35.7% (11, 12). Bai R and his colleagues reviewed 40 cases of GFAP-A patients first diagnosed with CNS infection, of which 17 were misdiagnosed as TBM cases (13). Lan W et al. conducted a detailed comparison of TBM and GFAP-A symptoms and discovered that myelitis, peripheral neuropathy, and movement disorders are significantly more prevalent in GFAP-A (7). Additionally, imaging has shown that lesions in GFAP-A extend beyond the meninges, with radial periventricular involvement being a distinctive feature (2). Thus, in patients presenting with meningitic symptoms and TBM-like CSF profiles but negative pathogen tests and nonspecific imaging, GFAP-A should be considered, warranting early GFAP-IgG testing.

Patient B exhibits a greater degree of symptomatology and a greater number of lesions on imaging than Patient A does. In diagnosing Patient B, neurological autoimmune disorders are prioritized alongside TBM. First, the diverse symptoms of Patient B, such as limb weakness, numbness, double vision, dizziness, and tremors, complicated our diagnosis. His MRI findings suggested changes in the brain and spinal cord, and the EMG results pointed to peripheral nerve damage, which led us to suspect an immune-mediated disease. The loss of vision particularly suggested the possibility of neuromyelitis optica spectrum disorder (NMOSD). On this basis, we conducted specific antibody tests for autoimmune encephalitis (AE) and paraneoplastic syndrome (PS) as much as possible. According to previous studies, one-third of GFAP-A patients exhibit paraneoplastic phenomena, with ovarian teratomas being the most common type of tumor (6). Finally, GFAP-IgG was identified, with the CSF titer significantly exceeding the serum titer. No abnormalities were detected for the other antibodies (14). In an observational study, Deng B et al. reported that peripheral neuropathies in patients with GFAP-A mostly affect the nerves in the legs and are linked to long-term disability (15).

Patient C presented a diagnostic challenge due to the absence of overt abnormalities upon examination. Despite this, he experienced significant distress from bilateral lower limb weakness and gait instability. Given the early onset and lack of additional comorbidities, an initial diagnosis strongly suggested a correlation with spinocerebellar ataxia (SCA) in Patient C. However, comprehensive genetic testing failed to reveal any anomalies in the genes typically associated with SCA. We must explore the possibility of immune-mediated cerebellar ataxias. The geometry of the cerebellar circuits is distinctive and characterized by a high density of neurons and glial cells that are enriched with ion channels and receptor proteins. This particular configuration renders them susceptible to immune disorders (16). Following comprehensive autoimmune antibody screening, we eventually detected GFAP antibodies in both the CSF and serum. A substantial cohort study conducted in Japan on individuals with GFAP-A revealed that movement disorders are relatively common in this population, with ataxia accounting for approximately 49% of cases (17). Although previous reports have documented the ataxia of patients with GFAP-A, ataxia is rare. In addition, Iorio R et al. reported that 3 GFAP-A patients with ataxia had coexisting autoantibodies (11). Therefore, further relationships between GFAP-IgG and ataxia need to be discussed.

## 4 Conclusion

Therefore, we present three illustrative cases characterized by symptoms involving multiple perspectives, with the objective of assisting clinicians in making a more accurate differential diagnosis. GFAP-A often manifests with a clinical presentation that mimics a variety of other diseases. This is attributable not only to the broad spectrum of lesions associated with GFAP-A but also to the presence of concurrent disease processes. Similarly, there is often delayed onset of brain magnetic resonance imaging abnormalities in GFAP-A (18). In alignment with the treatment protocols for most neuroimmune disorders, the therapeutic approach for GFAP-A primarily includes the administration of corticosteroids, intravenous immunoglobulin, and plasma exchange (19). Furthermore, GFAP-A is often associated with paraneoplastic tumors, underscoring the need for long-term follow-up in this patient population.

## Data Availability

The raw data supporting the conclusions of this article will be made available by the authors, without undue reservation.
